# *In Vitro* Antioxidant Properties of Flavonoids and Polysaccharides Extract from Tobacco (*Nicotiana tabacum* L.) Leaves

**DOI:** 10.3390/molecules170911281

**Published:** 2012-09-21

**Authors:** Qiao-Mei Ru, Li-Juan Wang, Wei-Ming Li, Jing-Lu Wang, Yu-Ting Ding

**Affiliations:** College of Biological and Environmental Engineering, Zhejiang University of Technology, Hangzhou 310032, China

**Keywords:** tobacco (*Nicotiana tabacum* L.) leaves, flavonoids, polysaccharides, reducing power

## Abstract

In the present study, antioxidant properties of flavonoids and polysaccharides from tobacco (*Nicotiana tabacum* L.) leaves were evaluated in several *in vitro* systems, e.g., scavenging activities on hydroxyl, superoxide anion, 1,1-diphenyl-2-picrylhydrazyl (DPPH) and 2,2'-azino-bis(3-ethylbenzthiazoline-6-sulphonic acid) (ABTS) radicals, and reducing power. Flavonoids showed much better activity than polysaccharides in scavenging activities on free radicals. When compared to the positive control, ascorbic acid, both showed weaker antioxidant potential. However, flavonoids possessed comparable superoxide anion, DPPH and ABTS radical scavenging abilities to ascorbic acid at high concentration (600 μg/mL). Meanwhile, it was found that flavonoids had prominent effects on the reducing power, which was equivalent to ascorbic acid, and was significantly higher than polysaccharides. These results clearly indicate that flavonoids are effective in scavenging free radicals and have the potential to be powerful antioxidants. Thus, tobacco leaves could be considered as a potential source of natural antioxidants for food, pharmaceutical, cosmetics or nutraceutical industries.

## 1. Introduction

In China, tobacco (*Nicotiana tabacum* L.) is a very important economic crop. Tobacco leaf production in China was about 2.5 million tons in 2008, representing an increase in production of 22.9% compared with that in 2007 [1]. However, over 20% of tobacco resources are discarded as processing waste, which pollutes the environment and cause a big waste issue. In fact, the discarded tobacco leaves are economically valuable because of abundant bioactive compounds in them, such as polyphenols, proteins and aromatic compounds [1,2,3]. Therefore, it is important to investigate and better utilize the tobacco leaves resource. Wang *et al.* [4] identified the polyphenols in tobacco leaves and their antioxidant and antimicrobial activities were also investigated. 

Flavonoids, very important constituents of plants, may contribute directly to anti-oxidative action because of the scavenging ability conferred by their hydroxyl groups. It was well document that polyphenolic compounds have inhibitory effects on mutagenesis and carcinogenesis in humans when up to 1 g daily is consumed from a diet rich in fruits and vegetables [5]. Interest in phenolics is increasing in the food industry because of their ability to retard oxidative degradation of lipids, thereby improving the quality and nutritional value of foods [6]. Polysaccharides are composed of 100 or more monosaccharides and often present in plants. As naturally occurring biological constituents, these high molecular weight polymers are highly appreciated for their multipurpose therapeutic properties, such as antitumor, immune-modulating, anti-flammatory, anti-pathogens, and antioxidant activities [7]. Thus, it would be beneficial to isolate polysaccharides from tobacco leaves for using as a nutritional supplement. Therefore, the basic aim of this research was to study antioxidant properties of flavonoids and polysaccharides from tobacco leaves.

## 2. Results and Discussion

### 2.1. Flavonoids and Polysaccharides from Tobacco Leaves

The yields of flavonoids and polysaccharides from tobacco leaves on a dry weight basis were 10.83 ± 0.91 mg RE/g and 49.82 ± 3.42 mg/g, respectively. For the polysaccharidic fraction, a level of 46.61 ± 3.11 mg/g was shown for the neutral polysaccharide, whereas 3.21 ± 0.22 mg/g was found for the acidic polysaccharides. The total phenolic contents of the flavonoid and polysaccharidic fractions were investigated, which were 23.2 ± 1.31 and 0.74 ± 0.04 mg GAE/g, respectively. It was confirmed that some polysaccharides are linked to phenols, and might be co-extracted using polar solvents [8]. Wang *et al.* [4] characterized rutin and chlorogenic acid as the two main polyphenol compounds in tobacco leaves. 

### 2.2. Scavenging Ability on Hydroxyl Radicals

**Figure 1:**
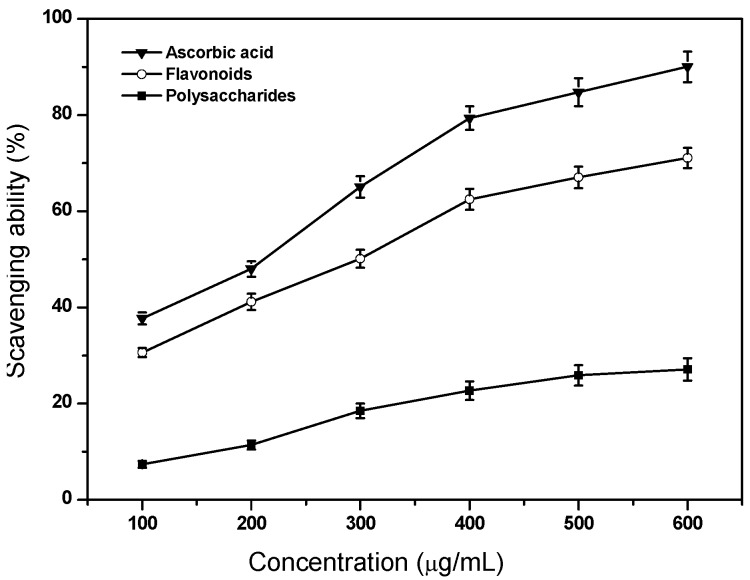
Scavenging ability of flavonoids and polysaccharides of tobacco leaves on hydroxyl radicals. Data are means ± standard deviation of triplicate experiments.

The scavenging effects of flavonoids and polysaccharides on hydroxyl radicals are shown in Figure 1. It was noticed that ascorbic acid showed the most pronounced effect, followed by flavonoids and polysaccharides. The present results proved that flavonoids was a good scavenger for hydroxyl radicals. Wang *et al.* [9] reported that a rutin standard showed much higher inhibition effect than acidic flavonoids and neutral flavonoids from *Lycium barbarum* L. The high antioxidant activity of flavonoids can be attributed to hydroxy groups in the A- and B-rings in rutin, and the greater the number of hydroxy groups, the higher is the capacity to scavenge free radicals [10]. Moreover, neutral polysaccharides exhibited much poor scavenging effect than acid polysaccharides in *L. barbarum* L. [9]. 

### 2.3. Scavenging Ability on Superoxide Anion Radicals

**Figure 2:**
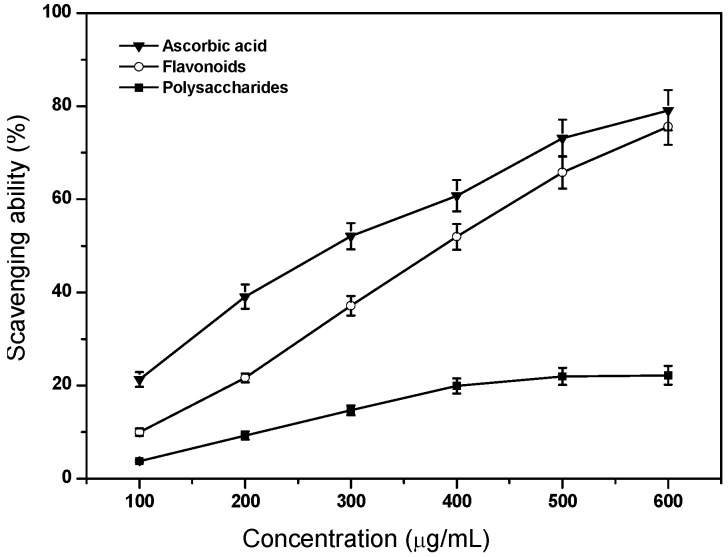
Scavenging ability of flavonoids and polysaccharides of tobacco leaves on superoxide anion radicals. Data are means ± standard deviation of triplicate experiments.

The effects of flavonoids and polysaccharides on superoxide anion radicals were determined and the results are shown in Figure 2. For the control treatment, the scavenging effect of ascorbic acid was substantially higher than those of flavonoids and polysaccharides (*p* < 0.05). However, flavonoids possessed comparable radical scavenging abilities with ascorbic acid when the tested concentration was at 600 μg/mL (*p* > 0.05). Flavonoids were effective scavengers for superoxide anion radicals, and could be advantageous for preventing injury induced by superoxide radicals in pathological conditions. A similar phenomenon was also observed in *L. barbarum* L. [9]. For flavonoids, the keto group at C-4' and hydroxy group at C-3' or C-5' of the C-ring were effective in scavenging superoxide anion, and the greater the number of hydroxy groups, the better is the scavenging activity [11]. Meanwhile, the hydroxy groups in the A- and B-rings in rutin should contribute to this effect due to their hydrogen donation ability [12]. 

### 2.4. Scavenging Ability on DPPH Radicals

**Figure 3:**
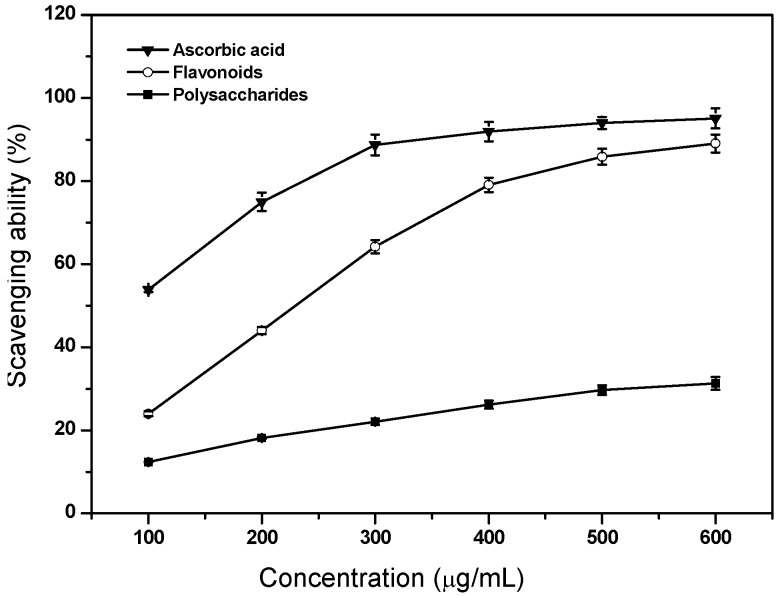
Scavenging ability of flavonoids and polysaccharides of tobacco leaves on DPPH radicals. Data are means ± standard deviation of triplicate experiments.

Figure 3 shows the scavenging effects of flavonoids and polysaccharides on DPPH free radicals. As expected, flavonoids exhibited a strong ability to quench DPPH radicals, and the DPPH radical scavenging activity of flavonoids at high concentration (600 μg/mL) was parallel to that of ascorbic acid (*p* > 0.05). This indicated that flavonoids were good antioxidants with strong DPPH radical scavenging activity. This result is in good agreement with that of Dziri *et al.* [13], who reported that the antioxidant activity of rosy garlic in the DPPH test was mainly attributable to flavonoids. In *Ganoderma lucidum* from Northeastern Portugal, a high linear correlation between the DPPH scavenging activity of phenolic extracts and total phenolic contents were demonstrated [14]. The acidic polysaccharides showed a larger DPPH free radical-scavenging activity than did the neutral polysaccharides in *L. barbarum* L. [9], which should be due to the ability of galacturonic acid present in the former to chelate metal ion and in turn scavenge DPPH radical. Asker *et al.* [15] isolated acidic polysaccharide from *Bacillus polymyxa* NRC-A and found that the antioxidant activity of polysaccharide was correlated with the content of galacturonic acid.

### 2.5. Scavenging Ability on ABTS Radicals

**Figure 4:**
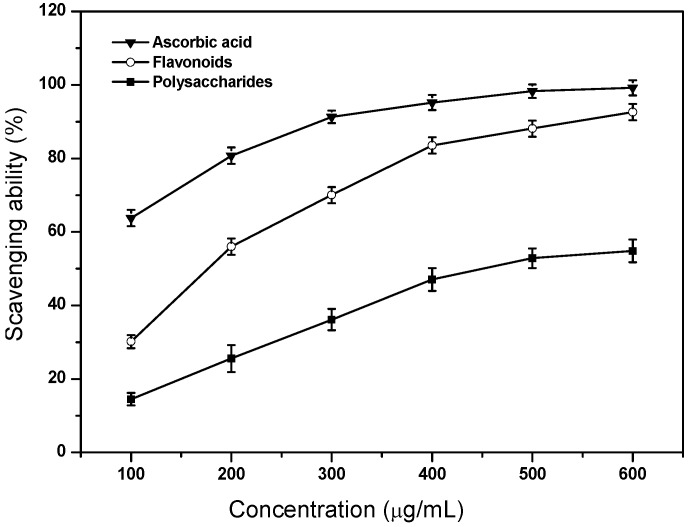
Scavenging ability of flavonoids and polysaccharides of tobacco leaves on ABTS radicals. Data are means ± standard deviation of triplicate experiments.

Figure 4 reveals the scavenging abilities of flavonoids and polysaccharides of tobacco leaves on ABTS radicals. As shown for ABTS scavenging, these data indicated the higher capacity of flavonoids to quench ABTS as compared to the polysaccharides (*p* < 0.05), and was parallel to ascorbic acid at high concentration (600 μg/mL, *p* > 0.05). These results are consistent with those of Wang *et al.* [9]. According to Oszmianski *et al.* [16], the antioxidant activities against DPPH or ABTS radicals were correlated with the concentration, chemical structures, and polymerization degrees of organ antioxidants. 

### 2.6. Reducing Power Ability

**Figure 5:**
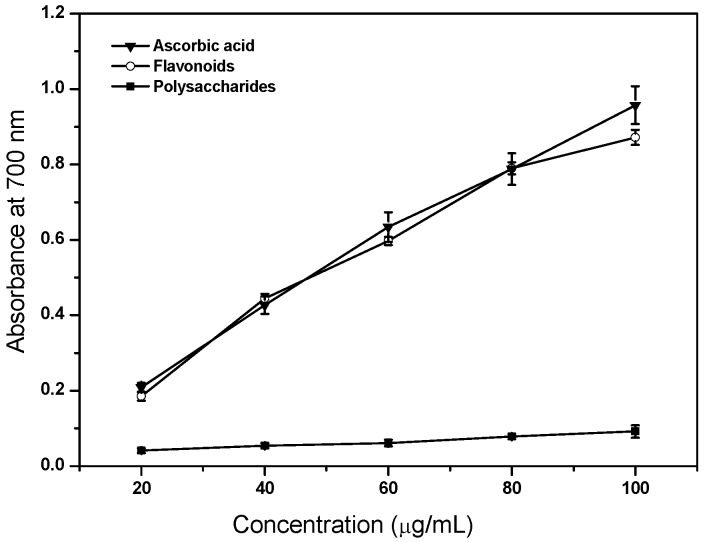
Reducing power of flavonoids and polysaccharides of tobacco leaves. Data are means ± standard deviation of triplicate experiments.

Figure 5 describes the reducing power of flavonoids and polysaccharides of tobacco leaves. As can be seen, flavonoids showed the same antioxidant property as ascorbic acid (*p* > 0.05), indicating that flavonoids were effective as an antioxidant. High reducing power of flavonoids suggested their remarkable potency to donate electrons to reactive free radicals, thus converting them into more stable non-reactive species and finally terminate the free radical chain reaction [17]. However, polysaccharides were still less effective than flavonoids (*p* < 0.05). Our data is consistent with the previous study of Wang *et al.* [9]. It was confirmed that the hydroxy groups at C-3' and C-4' of the B-ring to be more active in reducing iron concentration [18]. In this study, the flavonoid fraction contained rutin, which belong to the flavonols and the presence of a catechol group in the B-ring should contribute to the reducing power of tobacco leaves. In *G. lucidum* from Northeastern Portugal, the low linear correlation between the reducing power and total polysaccharides content was provided, whereas considering phenolic contents obtained in polysaccharidic extract, the linear correlation with antioxidant activity increased [14]. 

### 2.7. EC_50_ Values for Antioxidant Properties

Concentration of sample at which the inhibition percentage reaches 50% is the EC_50_ value. The EC_50_ values of flavonoids and polysaccharides for free radical scavenging activities, as well as reducing power abilities, are summarized in Table 1. EC_50_ values of flavonoids for scavenging activities on hydroxyl, superoxide anion, DPPH and ABTS radicals were 305, 390, 235 and 175 μg/mL, respectively, which were lower than those of polysaccharides (all > 600 μg/mL). The EC_50_ value of flavonoids (47 μg/mL) for reducing power was similar with the control, ascorbic acid, and was significantly higher than that of polysaccharides (>100 μg/mL). Our results agreed with the findings of Ramos *et al.* [19] who found that the antioxidant activity of onion extracts was mainly attributable to flavonoids.

**Table 1:** EC_50_ values of flavonoids and polysaccharides of tobacco leaves.

Test	EC_50_ value (μg/mL)		
	Flavonoids	Polysaccharides	Ascorbic acid
Scavenging ability on hydroxyl radicals	305	>600	210
Scavenging ability on superoxide radicals	390	>600	285
Scavenging ability on DPPH radicals	235	>600	>100
Scavenging ability on ABTS radicals	175	>600	>100
Reducing power	47	>100	47

EC_50_ value is negatively related to the antioxidant activity, as it expresses the amount of antioxidant needed to decrease the radical concentration by 50%. The lower the EC_50_ value, the higher is the antioxidant activity of the tested sample. Sun *et al.* [5] have reported the antioxidant properties of flavonoids from persimmon (*Diospyros kaki* L.) leaves, a traditional Chinese medicine, and indicated that the EC_50_ values for hydroxyl, superoxide and DPPH radical-scavenging ability were 70.64 μg/mL, 41.58 μg/mL and 96.36 μg/mL, respectively, which were much lower than that of flavonoids from tobacco leaves. However, the EC_50_ values for hydroxyl and superoxide anion radical-scavenging ability of flavonoids obtained by an ethanol extraction method from sweet potato (*Ipomoea batatas* L.) leaves, one of the most important economic crops, were about 0.8 mg/mL and 1.2 mg/mL, respectively [20], indicating much weaker antioxidant ability than the flavonoids in this experiment. In addition, with regard to reducing power, the flavonoids showed higher ability than phenolic and polysaccharidic extracts of a popular medicinal mushroom, *G. lucidum* fruiting body, which were 0.62 mg/mL and 0.81 mg/mL, respectively. Therefore, it was indicatative that flavonoids from tobacco leaves are effective in scavenging free radicals and have the potential to be a powerful antioxidant.

## 3. Experimental

### 3.1. Materials and Chemicals

Tobacco leaves were provided by China Tobacco Gansu Industrial Co., Ltd., dried at 70 °C in an oven and ground to obtain fine powder (50 mesh). Ascorbic acid, ABTS, DPPH, chlorogenic acid (95% purity) and rutin (95% purity) were purchased from Sigma Chemical Co. (St. Louis, MO, USA). All other chemicals were analytical grade and purchased from Shanghai Boer Chemical Reagent Co., Ltd. (Shanghai, China).

### 3.2. Isolation of Polysaccharides

The polysaccharides of tobacco leaves was isolated according to the method described by He *et al.* [21] and Zhang *et al.* [22] with a minor modification. Tobacco leaves powder (50 g) was extracted with 20 volumes of distilled water at 100 °C for 2 h. The suspension was centrifuged (8,000 × *g*) for 15 min, and the supernatant was concentrated under vacuum. The protein in the product of condensation was deproteinized using the Sevag reagent [23]. After removal of the Sevag reagent, four volumes of anhydrous ethanol were added to this concentrate, and the mixture was stirred at room temperature for 20 min and then left at 4 °C overnight. The precipitate was collected by centrifugation (8,000 × *g*) for 15 min, subsequently washed three times with anhydrous ethanol, acetone and ether, respectively, then dissolved in distilled water and dialyzed in dialysis bag (molecular weight cut-off, 12 kDa) against distilled water at room temperature for three successive days. The retained fraction was recovered, concentrated under vacuum and lyophilized to obtain crude polysaccharide. Crude polysaccharide was dissolved in distilled water, centrifuged, and then the supernatant was applied to a column of anion exchange chromatography on DEAE-Sepharose Fast Flow column (100 × 2.6 cm^2^), eluting with 0–2 M gradient NaCl solution. The fractions eluted with 0–2 M gradient NaCl were collected, concentrated, dialyzed and lyophilized to get the purified polysaccharides. 

### 3.3. Quantitation of Polysaccharide Content

The content of polysaccharides was determined using the phenol–sulphuric acid method [24]. Briefly, polysaccharide solution (0.5 mL) was mixed with phenol solution (0.5 mL, 5%, w/v), followed by addition of concentrated sulphuric acid (2.5 mL) and shaking the mixture for 50 min. The absorbance was measured at 490 nm and used to quantify polysaccharides, based on the standard curve of glucose (10–1,000 μg/mL).

### 3.4. Isolation of Flavonoids

Dried tobacco leaves powder (50 g) was placed in a reflux apparatus. Extraction was performed with 85% (v/v) aqueous ethanol (500 mL) for 60 min at 80 °C. The crude extract was filtered. The solution was concentrated under reduced pressure. The crude flavonoids-enriched extract was purified using a column (25 × 1.5 cm^2^) packed with AB-8 macroporous adsorption resin according to the reference [25]. The conditions for purifying the flavonoids by AB-8 resin were: injecting concentration 3.75 mg/mL, pH = 5, injecting velocity 2.0 mL/min, 40% (v/v) ethanol as desorption solvent, desorption velocity of flow 1.5 mL/min. The purified extract of flavonoids was collected and evaporated at 50 °C, and was then freeze-dried for determination of flavonoid content and antioxidant property.

### 3.5. Determination of Flavonoid Content

The content of flavonoids was determined using a colormetric method described by Yi *et al.* [26] with several modifications. Briefly, diluted sample (1 mL) was mixed with NaNO_2_ (1 mL, 5%, w/v). After 6 min, AlCl_3_ (1 mL, 10%, w/v) was added and allowed to stand for 6 min, then NaOH (5 mL, 4%, w/v) was added to the mixture. Absorbance was taken at 510 nm after 15 min. The content of flavonoids was expressed as rutin equivalents determined from a rutin calibration curve (10–1,000 μg/mL).

### 3.6. Determination of Total Phenolic Content

Total phenolic content was measured by using the Folin-Ciocalteu reagent according to the method described [27] and using gallic acid as standard, with some modifications. Briefly, diluted extract (0.5 mL) was mixed with freshly diluted 10-fold Folin-Ciocalteu reagent (5.0 mL). The mixture was allowed to react for 3 min and 7% aqueous solution of Na_2_CO_3_ (4.0 mL) was added. The reaction mixture was kept in the dark for 2 h intermittent shacking and the absorbance was read at 760 nm. 

### 3.7. Assay of Antioxidant Property

#### 3.7.1. Scavenging Activity on Hydroxyl Radicals

Hydroxyl radical scavenging activity was determined based on the method described by Smirnoff and Cumbes [28] with some modifications. The reaction mixture containing the tested sample (1 mL) was incubated with a solution containing orthophenanthroline (1 mL, 5 mM), phosphate buffer (0.8 mL, 7.5 mM, pH 7.4) and FeSO_4_ (0.5 mL, 7.5 mM). Finally, H_2_O_2_ (0.5 mL, 8.8 mM) was added, and the reaction mixture was then incubated at 37 °C for 1 h. The absorbance of the resulting solution was measured spectrophotometrically at 532 nm. The hydroxyl radical scavenging ability was calculated using the following formula: Scavenging ability (%) = (1 − A_sample_/A_control_) × 100, where A_control_ is the absorbance of control without the tested sample, and A_sample_ is the absorbance in the presence of the tested sample. The tested sample concentration providing 50% inhibition (EC_50_) was calculated from the graph of scavenging effect percentage against tested sample concentration. Ascorbic acid was used as reference compound.

#### 3.7.2. Scavenging Activity on Superoxide Anion Radicals

The scavenging activity of superoxide anion radicals was assessed referring to the reference [29] with several modifications. A tube containing Tris-HCl buffer (3 mL, 50.0 mM, pH 8.2) and tested sample (1 mL) was incubated in a water bath at 25 °C for 20 min, then pyrogallic acid (0.4 mL, 5.0 mM) at the same temperature was added and proceed at 25 °C. HCl solution (0.1 mL, 8.0 M) was used to terminate the reaction after 4 min. The absorbance of the mixture was measured at 320 nm. The scavenging ability of superoxide anion radicals and EC_50_ were calculated as Section 3.6.1. For comparison, ascorbic acid was used as positive control.

#### 3.7.3. Scavenging Activity on DPPH Radicals

DPPH radical scavenging activity was measured according to Braca *et al.* [30]. Tested sample (0.2 mL) was added to methanolic DPPH solution (2.8 mL, 0.1 mM), then shaken vigorously and left standing at room temperature for 30 min in the dark. The absorbance of the resulting solution was then measured at 517 nm. The DPPH radical scavenging effect and EC_50_ were calculated as Section 3.6.1. Ascorbic acid was used for comparison.

#### 3.7.4. Scavenging Activity on ABTS Radicals

The scavenging activity against ABTS radicals (ABTS^+^) was measured using the method of Fellegrini *et al.* [31] with some modifications. ABTS^+^ were produced by reacting ABTS solution (7 mM) with potassium persulphate (1.4 mM), and the mixture would be kept in the dark at room temperature for 12~16 h. In the moment of use, the ABTS^+^ solution was diluted with ethanol to an absorbance of 0.70 ± 0.02 at 734 nm. Tested sample (0.2 mL) was added to ABTS^+^ solution (2.8 mL) and mixed vigorously. After reaction at room temperature for 6 min, the absorbance at 734 nm was measured. The ABTS radical scavenging effect and EC_50_ were calculated as Section 3.6.1. Ascorbic acid was used as reference compound.

#### 3.7.5. Reducing Power Ability

The reducing power was assessed referring to the method described in literature [32] with slight modifications. Tested sample (1 mL) was mixed with sodium phosphate buffer (2.5 mL, 0.2 M, pH 6.6) and K_3_Fe(CN)_6_ (2.5 mL, 1%, w/v), and the mixture was incubated at 50 °C for 20 min. After that, TCA (2.5 mL, 10%, w/v) were added, and the mixture was centrifuged at 3,000 × *g* for 10 min. Upper layer fraction (2.5 mL) was mixed with deionized water (2.5 mL) and FeCl_3_ (0.5 mL, 0.1%, w/v) and thoroughly mixed. The absorbance was measured at 700 nm and ascorbic acid was used as positive control. A higher absorbance indicates a higher reducing power. The tested sample concentration providing 0.5 of absorbance (EC_50_) was calculated from the graph of absorbance registered at 700 nm against the correspondent tested sample concentration.

### 3.8. Statistical Analysis

In order to assure the accuracy of the experimental data, each experiment was performed in triplicate and the result was expressed as mean ± standard deviation of three replications. *p* vaule < 0.05 was regarded as significant.

## 4. Conclusions

Scavenging activities on free radicals and reducing power were investigated for flavonoids and polysaccharides of tobacco leaves. The flavonoids showed the more pronounced effect in antioxidant activity than polysaccharides. From the above results, it appears important to develop natural antioxidants from tobacco leaves, and this may be a good way for extensively utilizing the tobacco resource.
